# Increased Biomass, Seed Yield and Stress Tolerance Is Conferred in *Arabidopsis* by a Novel Enzyme from the Resurrection Grass *Sporobolus stapfianus* That Glycosylates the Strigolactone Analogue GR24

**DOI:** 10.1371/journal.pone.0080035

**Published:** 2013-11-05

**Authors:** Sharmin Islam, Cara A. Griffiths, Cecilia K. Blomstedt, Tuan-Ngoc Le, Donald F. Gaff, John D. Hamill, Alan D. Neale

**Affiliations:** 1 School of Biological Sciences, Monash University, Melbourne, Victoria, Australia; 2 Biosciences Research Division, Victorian AgriBiosciences Centre, Melbourne, Victoria, Australia; 3 Department of Forest and Ecosystem Science, University of Melbourne, Creswick, Victoria, Australia; Ghent University, Belgium

## Abstract

Isolation of gene transcripts from desiccated leaf tissues of the resurrection grass, *Sporobolus stapfianus*, resulted in the identification of a gene, *SDG8i*, encoding a Group 1 glycosyltransferase (UGT). Here, we examine the effects of introducing this gene, under control of the CaMV35S promoter, into the model plant *Arabidopsis thaliana*. Results show that *Arabidopsis* plants constitutively over-expressing *SDG8i* exhibit enhanced growth, reduced senescence, cold tolerance and a substantial improvement in protoplasmic drought tolerance. We hypothesise that expression of *SDG8i* in *Arabidopsis* negatively affects the bioactivity of metabolite/s that mediate/s environmentally-induced repression of cell division and expansion, both during normal development and in response to stress. The phenotype of transgenic plants over-expressing *SDG8i* suggests modulation in activities of both growth- and stress-related hormones. Plants overexpressing the UGT show evidence of elevated auxin levels, with the enzyme acting downstream of ABA to reduce drought-induced senescence. Analysis of the *in vitro* activity of the UGT recombinant protein product demonstrates that SDG8i can glycosylate the synthetic strigolactone analogue GR24, evoking a link with strigolactone-related processes *in vivo*. The large improvements observed in survival of transgenic *Arabidopsis* plants under cold-, salt- and drought-stress, as well as the substantial increases in growth rate and seed yield under non-stress conditions, indicates that overexpression of *SDG8i* in crop plants may provide a novel means of increasing plant productivity.

## Introduction

 The desiccation tolerant grass *Sporobolus stapfianus* grows in shallow, nutrient poor soils in regions experiencing intense seasonal drought. For their persistence these plants rely on the ability of the protoplasm of their vegetative tissue to desiccate (loss of ≥ 95% total water content) and rehydrate rapidly. The rehydrated plant restores normal metabolism within 24 hours [1], grows very quickly following rain, and has proven useful for pinpointing genes for increased stress-tolerance [2,3] and enhanced growth rate [4]. Characterization of *Sporobolus* drought genes (*SDG*s) that are specifically expressed in desiccation-tolerant tissue [5], has the potential to reveal mechanisms for coping with stress which are peculiar to, or enhanced in, resurrection plants. Such coping mechanisms include the ability to adjust growth rapidly in response to changes in water availability, to inhibit dehydration-induced senescence programs, to protect cellular components during dehydration and to reinstitute photosynthetic capacity quickly following a severe dehydration event [1]. The mechanisms required for *S. stapfianus* to exhibit these characteristics may rely on coordinately regulated plant hormone activity linked to environmental cues. 

 The *Sporobolus SDG8i* gene encodes a Group 1 UDP-glycosyltransferase (UGT) whose transcript levels increase substantially under severe water deficit [5]. Plant genomes typically encode a large number of UGTs that collectively can conjugate sugars to a range of acceptor molecules including many plant hormones, secondary metabolites and xenobiotics [6]. UGTs have an important role in cellular metabolism since glycosylation can affect the solubility, transport and biological activity of these compounds [7]. Hence glycosylation can control the bioactivity of plant growth regulators crucial to enabling adaption of plants to changing environments [8]. The majority of the classical hormones occur as glycosides *in planta* and UGTs capable of glycosylating auxins, cytokinin, ABA, salicylic acid, jasmonic acid and brassinosteroids or their synthetic precursors have been identified [9-15]. The possibility that glycosylation of one or more growth regulators may play a role in promoting onset of desiccation tolerance in *S. stapfianus* was suggested by the study of Le et al. [5],, but as yet no experimental evidence for such a role has been reported. 

 As no protocol for transformation of resurrection grasses exists, functional analysis of the dehydration-induced UGT SDG8i was undertaken in *Arabidopsis*. Ectopic expression of SDG8i in *Arabidopsis* was found to have a profound effect on plant architecture and growth and confer a substantial improvement in protoplasmic drought tolerance. Here we report that *SDG8i* encodes a functional UGT that can glycosylate the synthetic strigolactone analogue GR24, and that ectopic expression of this UGT leads to a substantial enhancement of plant growth and stress resistance.

## Materials and Methods

### Plant materials and growth conditions

*Arabidopsis thaliana* (L.) Heynh, *Sporobolus stapfianus* Gandoger and *Sorghum bicolor* L. seed were obtained from laboratory stocks. Wild-type (WT) plants refer to *Arabidopsis* accession Columbia-0 (Col-0). *Orobanche* seeds were obtained from the South Australian Department of Water, Land and Biodiversity Conservation. *Arabidopsis* plants were stratified at 4°C for 3 days and grown at 22°C under continuous light unless stated otherwise. Under long day (LD) photoperiod conditions the plants were subjected to a 16 hour light and 8 hour dark cycle. Under a short day (SD) photoperiod, the cycle consisted of 8 hours light and 16 hours dark. Soil grown plants were placed in a growth cabinet at 22°C, 25% relative humidity and approximately 200 µmole/m^2^/sec light intensity. For axenic culture, seeds were surface-sterilized in 70% (v/v) ethanol and rinsed with sterile water and cultured at 22°C with approximately 100 µmole/m^2^/sec light intensity. Crossing of *Arabidopsis* plants was performed as described in Weigel and Glazebrook [16].

### Generation of transgenic plants

 The *SDG8i* coding sequence (EMBL/GenBank accession number AM268210) was amplified and inserted into the donor vector pDONR221 using the Gateway cloning system (Invitrogen) following the manufacturer’s instructions.

5′attB1 Primer;
GGGGACAAGTTTGTACAAAAAAGCAGGCTATGACGAAGACCGTGGTTCTG
3′attB2 Primer;
GGGGACCACTTTGTACAAGAAAGGTGGGTCTCACGGACGACCGACAGCCTCCA

The pDONR221:SDG8i construct was transferred to the expression vector pMDC32 containing the 2 × 35S promoter [17] and transformed into *Arabidopsis* Columbia-0 (Col-0) using *Agrobacterium tumerfaciens* (AGL-1strain) by the floral dip method [18]. Second generation (T2) transgenic plants homozygous for *SDG8i* were generated under hygromycin resistance.

### Recombinant UGT production

 The UGT was produced by transient transformation of *Nicotiana bethamiana* leaves using a viral MagnICON vector system (Icon Genetics GmbH, Germany). The *SDG8i* sequence was amplified by RT-PCR, using RNA from *S. stapfianus*, with the primers:

Forward: 5′GAGAGAATTCATGACGAAGACCGTGGTTCTGTAC3 ′ 

Reverse: 5′GAGAGGATCCTCACGGACGACCGAC3 ′. 

 The PCR product was ligated into pICH11599 to generate pICH11599-*SDG8i*. The three pro-vector fragments: (i) pICH12190 (containing a chloroplast signal sequence), (ii) pICH11599-*SDG8i* and (iii) pICH14011 (the integrase) were separately electroporated into *Agrobacterium tumerfaciens* GV3101. For the vector-only control pICH11599 was used with (i) and (iii). Equal amounts of the three *A. tumerfaciens* strains were infiltrated into aluminum foil-covered *N. benthamiana* leaves using the protocol described by Marillonet et al. [19]. After 5 days co-incubation, the leaf tissue was ground in liquid nitrogen and mixed with 500 µl cold protein extraction buffer (5mM sodium phosphate buffer pH 7.5, 10mM EDTA, 0.1% (v/v) Triton X-100). The extract was centrifuged (13,500 g) for 10 minutes at 4°C and the protein content was determined by Bradford assay and extracts analysed by SDS-PAGE [20].

### Observation of palisade cells in fully expanded leaves

 The second fully expanded rosette leaves from transgenic and control seedlings were immersed in 0.1% (v/v) Triton X-100, then centrifuged (10,000g) for 1 min to remove air from intercellular spaces and imaged using a light microscope. The leaf area was measured with Adobe Photoshop CS5.1 using the formula [(No. of pixels of leaves/No. of pixels of physical unit) × Area of physical unit]. Twenty sub-epidermal palisade cells aligned along the proximo–distal and medio–lateral axes were used to calculate the average cell area using ImageJ software. The total cell number per leaf was calculated by dividing the leaf area by the palisade cell area for each leaf.

### Examination of root architecture

 Plants were grown under SD (8h day/16h night) for 13 days in vertically-orientated petri dishes on MS media (pH 5.8) with 1% (w/v) sucrose and 0.8% (w/v) agar. Roots were then fixed in 4% (v/v) formaldehyde in 0.025 M phosphate buffer (pH 7.2) overnight at 4°C. The fixative was replaced with 30% (v/v) glycerol containing 2% (v/v) DMSO and left for 30 min at room temperature. Roots were mounted in a clearing solution (4.2 M NaI, 8 mM Na_2_S_2_O_3_, 2% (v/v) DMSO in 65% (v/v) glycerol) and root primordia and root cell-length were examined 1 h after the sample preparation. The number of primordia was determined within the lateral-root-formation zone between the most-distal initiated primordium and the most-distal emerged lateral root [21]. Lateral root primordium density (d) was calculated for each individual primary root as number of primordia per mm. *I*_LRI_, (the number of lateral root primordia initiated within a portion of the root that corresponds to the length (*l*, mm) of 100 fully elongated cortical cells in a single file in the same parent root) was determined as 100dl, where *l* is the average cortical cell length in mm for each individual primary root. 

### Histochemical analysis of ß-glucuronidase (GUS) activity

 Seedlings were GUS stained at 37°C for 4 h according to the protocol of Jefferson et al. [22] and then cleared with 70% (v/v) ethanol. Images were taken using a microscope (Leica MEFLIII).

### Salt stress assays

 Seedlings, grown for six days on vertically-orientated germination media plates (½ MS, 0.5% (w/v) sucrose, pH 5.7, 0.8% (w/v) agar), were transferred to fresh media containing salt (0, 50, 100, 125, 150 and 175 mM NaCl) and the position of root tips noted. The plates were inverted to allow roots to grow downward in the shape of a hook. Root elongation was measured after 7 days on salt media.

### Freezing stress assay

 Seedlings (25/plate) were grown for 3 weeks at 22°C on MS-agar (½ MS, 0.5% (w/v) sucrose, pH 5.7, 0.8% (w/v) agar) before acclimation at -1°C for 16 hours. The temperature was lowered by 2°C per hour. Seedlings were held at the desired temperature for one hour before a plate containing a subsample was removed, and then the temperature was lowered further. Following the one hour exposure to -4°C, -8°C and -12°C, subsampled plants were transferred to 4°C overnight for recovery then returned to 22°C and survival scored 7 days later. Plants that were bleached were scored as dead, while green plants were scored as having survived the freezing test.

### Water withholding test

 Plants in separate pots were kept fully and uniformly watered by sub-irrigation under optimal growth conditions in LD at 22°C until the 6-7 leaf pre-flowering stage. Water was then withheld, and the plants were observed over a period of 3 weeks. Subsamples of drought treated plants were re-watered at regular intervals and recovery was monitored after 24 hours. Five plants from each line were tested at each sample point and the experiment was performed twice. 

### Water vapor equilibration

 Protoplasmic drought tolerance (PDT), the water potential at which 50% of plant tissue survives in equilibrium with the air, was assessed using CaCl_2_ solutions. This permits water potentials (ψ_w_), down to 30% relative humidity (RH) in equilibrium with a saturated solution, to be imposed on the plants via the air phase [23]. The RH in equilibrium with the CaCl_2_ solutions is calculated from freezing point depression data [24]. Chambers containing CaCl_2_ solutions ranging from 98% (-2.8 MPa) to 86% (-20.5 MPa) RH at 25°C were used. Pre-flowering plants were soaked in water until turgid and then blotted dry. Shoots were detached and the initial turgid weight (TWt_o_) recorded. Shoots were enclosed in insulated chambers for 3 days until equilibration to differing RH was reached (ψ_leaf_ = ψ_solution_). The shoots were then rehydrated for 24 hours and the final turgid weight (TWt_F_) recorded. Survival of plants was determined by: Method A, the number of leaves alive per plant, judged subjectively by the recovery of the healthy coloration and crisp texture (dead leaves are readily distinguished in the thin fibre-poor tissues of *Arabidopsis*); Method B, an objective test, depends on the loss of semipermeability in dead cells which prevents osmotic absorption of water, resulting in an apparent loss in the “turgid” weight per dry weight of dead tissue compared with that of live tissue before vapor-equilibration. The ratio TWt_F_/TWt_o_ was plotted against % RH and the PDT determined as the % RH at which 50% of the tissue is alive. Four shoots from each line were tested for each sample point and the experiment was performed twice. 

### Senescence tests

 For dark-induced senescence, excised leaves from 12 day old soil-grown *Arabidopsis* plants were floated, adaxial side up, on deionized water. For ABA treatment, detached leaves were floated on 3 mM MES buffer (pH 5.8) solution under continuous light in the presence or absence of 50 µM abscisic acid. 

### Chlorophyll analysis

 Leaf tissue (20 mg) was ground under liquid nitrogen and extracted twice with 1.5 ml ice-cold 80% (v/v) acetone, centrifuged (14,000 rpm) at 4°C for 3 min and supernatants stored in the dark. The chlorophyll concentration was calculated as described by Lichtenthaler [25] . The F_v_/F_m_ ratio was measured after dark adaptation of the leaves for 15 min using a PAM-210 (Teaching-PAM) (Heinz Walz GmbH, Germany). 

### UGT enzyme assay

 Recombinant UGT activity was measured using the coupled enzyme assay described by Jackson et al., [9]. The assay was conducted at pH 7.4 with a final substrate concentration of 1 mM using 25 µg of protein extract. GR24 was obtained from Chiralix B.V. Nijmegen, The Netherlands. All other hormones were obtained from Sigma St. Louis, MO. Activity in millikatals kg^-1^ was calculated using the extinction coefficient 6.22 x 10^-3^ M^-1^ cm^-1^ for NADH. Background activity of extracts, monitored by measuring the rate without substrate addition, was subtracted from the reaction rate.

### *Orobanche* germination assay

*Orobanche* (*Phelipanche ramosa* (L.) Pomel) seeds were surface-sterilized with 2% sodium hypochlorite containing 0.02% (v/v) Tween-20, rinsed with sterile ddH_2_O and dried in a laminar flow cabinet. Seed (~100) on a sterile glass microfiber disk were placed on ddH_2_O soaked filter paper in Petri dishes and preconditioned by adding 1ml of GA_3_ (30 mg/L) and incubation at 20°C in the dark for 7 days. Surface-sterilized seeds of host plants were added to the microfiber disks and placed on ¼ MS media with 0.5% (w/v) sugar and 0.8% (w/v) agar. For controls, 0.6 ml of GR24 (0.0001 mg/L) or ddH_2_O was applied to the preconditioned *Orobanche* seeds. The plates were covered in foil and incubated at 20°C for 7 days. *Orobanche* germination was determined by counting the seeds with an emerged radicle.

### Effect of GR24 on shoot branching

To determine the effect of GR24 on shoot branching, *Arabidopsis* plants were grown in soil for 23 days to pre-bolting stage. The plants were then treated with 50 µl per plant of 5 µM GR24 applied to the shoot meristem and axillary meristem region. Control plants were treated with 50 µl of water. The treatment was repeated every third day for 20 days when the number of rosette branches (>5 mm) was counted.

### Statistical Analyses

 All data were examined by analyses of variance using GraphPad Prism software version 5.0. Tukey’s Multiple Comparison Test was used for comparison between means of wild-type and SDG8i transgenic plants at 5% level of significance.

## Results

### Ectopic expression of SDG8i affects the shoot architecture and the growth rate of *Arabidopsis*

**Figure 1:**
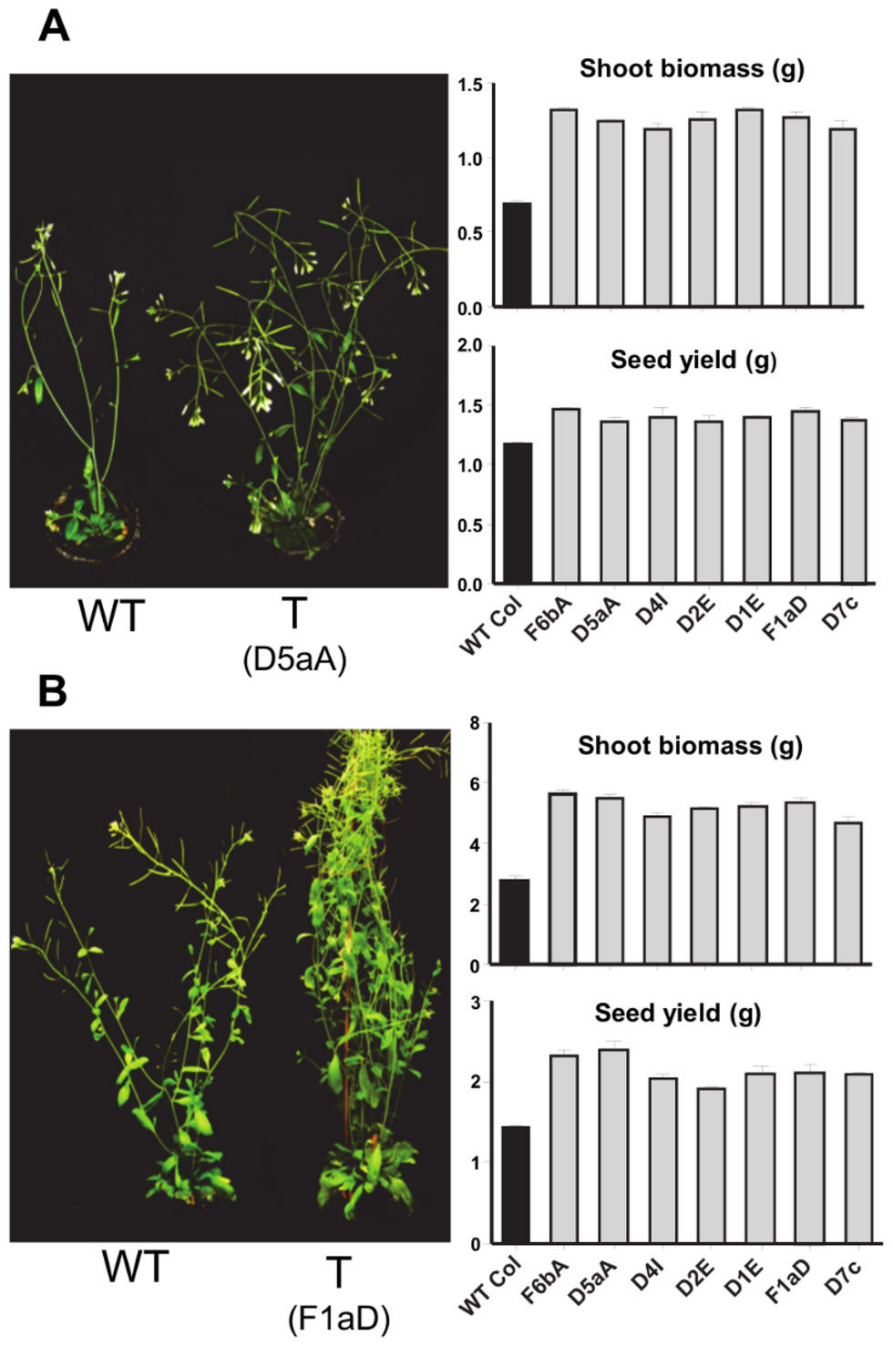
A phenotypic comparison of SDG8i transgenic (T) and wild-type Col-0 (WT) plants. Plants were grown at 21°C under (A) long day and (B) short day. The figure shows the increased height, branching, shoot biomass (FW) and seed yield typical of all the SDG8i transgenic lines. Dry seed was collected following senescence. The biomass was measured 16 days (LD) and 50 days (SD) after germination and before bolting. Values are the means ± SE of 5 replicates. The transgenic plant grown in short days has been tied with cotton thread.

 Several *Arabidopsis* lines (F1aD, D1E, F6bA, D5aA, D2E, D4I, D7c) designed to express the SDG8i protein under the control of the 35S promoter (SDG8i transgenic plants) were taken to homozygosity and the presence of the *SDG8i* transcript was confirmed (Fig. S1). The developmental phenotype of these independent SDG8i transgenic lines was examined under long day (LD: 16h day/8h night) and short day (SD: 8h day/16h night) photoperiods (Figure 1). Flower morphology, seed development and seed weight were normal (Fig. S2). Transgenic plants were not affected in flowering time in LD, with production of a similar number of primary rosette leaves as formed by the wild-type Col-0 controls. In SD, both wild-type Col-0 and transgenic plants produced the first inflorescence 52 days after germination, with wild-type plants producing around 30-34 primary rosette leaves compared with approximately 40-42 primary rosette leaves produced by the transgenics.

 When compared to wild-type plants the transgenic lines were taller, and had increased branching, shoot biomass and seed yield (*p*<0.05) (Figure 1 and Fig. S2 and S3). These phenotypic differences were more pronounced in SD. One month after flowering the total number of leaves, including primary rosette leaves and leaves formed in the rosette from axillary meristems by transgenic plants was 1.3 to 1.6 times more than wild-type plants (*p*<0.01) (Fig. S3A). The average size of the leaves of the transgenic plants was significantly larger than that of wild-type plants (*p*<0.05) (Fig. S3B) and the number of inflorescences produced was 1.4 to 1.6 times that of wild-type Col-0 (*p*<0.01) (Fig. S3C). Despite the increased branching, the height increases exhibited by SDG8i plants over wild-type ranged from 9% to 16% after 21 days of bolting (Fig. S3D). The rate of growth of the primary inflorescence of transgenic plants was approximately 20% greater (7.1 cm week^-1^) than that of wild-type plants (5.4 cm week^-1^). After 50 days in SD photoperiods the average fresh weight (FW) shoot biomass of all transgenic plants was almost twice that of wild-type (Figure 1) and the seed yield of SDG8i transgenic plants was 1.4-1.6 times the mass of seed from wild-type plants (*p*<0.001) (Figure 1). 

### The leaves of SDG8i plants have increased cell size and number

 The rosette leaves of SDG8i plants are larger than wild-type Col-0 plants in both LD and SD. When the leaf cells of SD soil-grown plants were examined, it was found that the leaves of SDG8i transgenic plants contained more cells than comparable leaves from control plants and the cells were larger (*p*<0.01) (Table 1 and Fig. S4). The increase in the number and size of leaf palisade cells of transgenic plants indicates that SDG8i activity promotes both leaf cell expansion and cell division and may affect the number of meristematic cells allocated to the leaf primordium.

**Table 1:** Leaf area, leaf cell size and leaf cell number in wild type (WT) and SDG8i plants.

**Plant Type**	**Av. Leaf Area**	**Av. Cell Area**	**Av. Cell**
	**(mm^2^)**	**(µm^2^)**	**Number/leaf**
WT	47.55 ± 1.98	3160 ± 30	15047 ± 586
D5aA	63.92 ± 0.84	3540 ± 159	18129 ± 845
D4I	62.0 ± 1.4	3575 ± 113	17392 ± 831
F6bA	59.97 ± 3.13	3592 ± 173	16858 ± 885

The leaf area and cell area were calculated in 25-day-old soil-grown plants in SD using imaging software. Values are the means ± SE of 3 replicates.

### SDG8i expression increases root cell length and lateral root initiation

**Figure 2:**
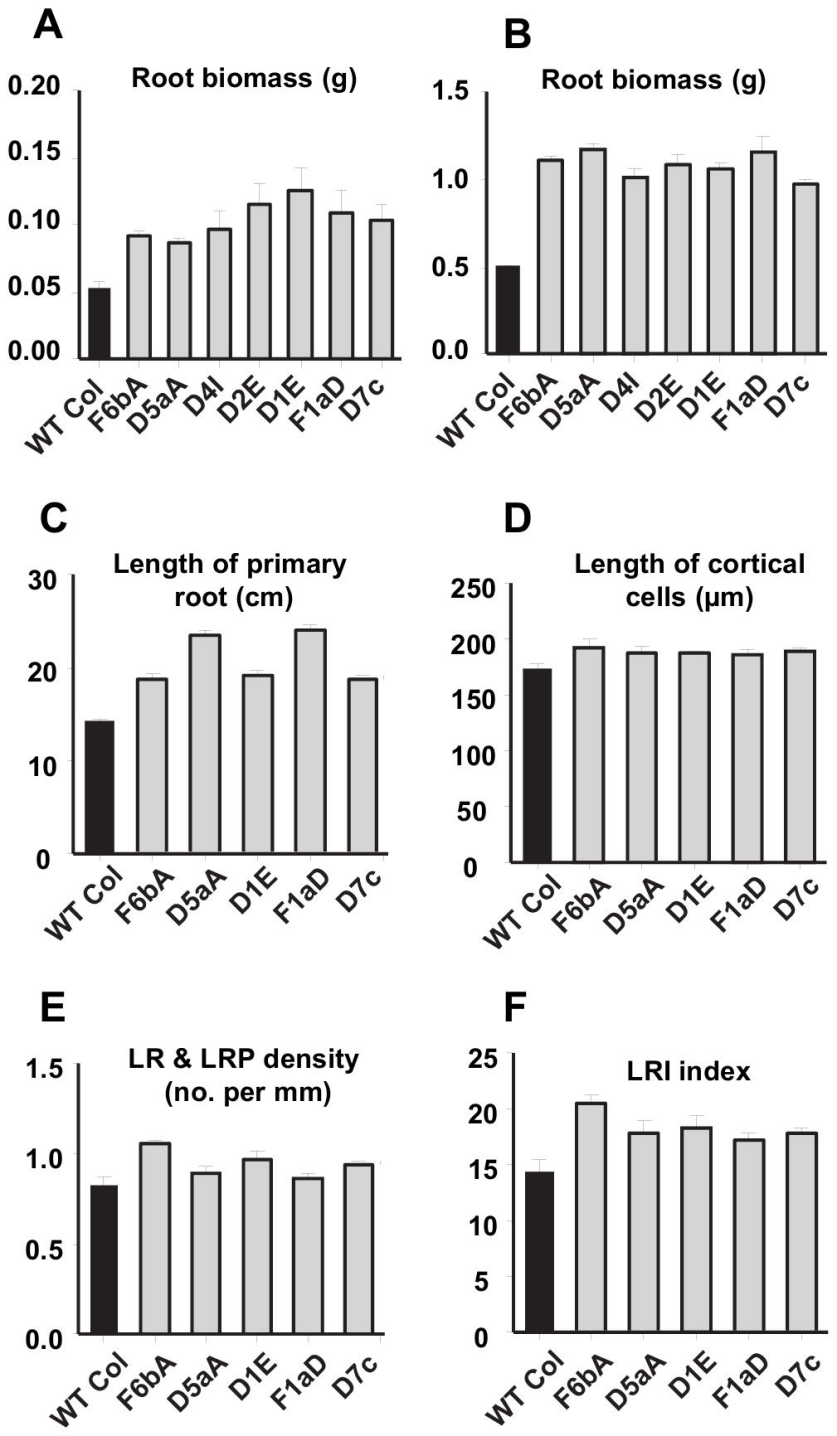
Root growth in SDG8i lines and wild-type Col-0 plants. Root biomass (FW) of pre-flowering plants growing in (A) long day at 21°C, measured 16 days after germination and (B) short day at 21°C, measured 50 days after germination. Values are the means ± SE of 4 replicates. Root development in 13-d-old *Arabidopsis* plants grown on vertical plates at 21°C under SD showing; (C) length of primary root and (D) length of fully elongated cortical cells and (E) lateral root primordium density and (F) lateral root initiation index. Values are the means ± SE of 4 replicates.

 When grown in both LD and SD the average fresh weight of the root biomass of all SDG8i transgenic plants was almost twice that of wild-type (Figure 2A, B). A comparison of the root architecture in SDG8i transgenics and wild-type plants grown *in vitro* in SD was conducted using the method described in Dubrovsky et al., [21]. The primary root of all SDG8i plants was longer than wild-type under SD (*p*<0.001) (Figure 2C). The fully elongated cortical cells in all the transgenic plants were longer than wild-type (*p*<0.05) (Figure 2D). Calculation of the lateral root primordium density also showed a difference between SDG8i transgenic plants and wild-type plants (*p*<0.005) (Figure 2E). The higher estimation of *I*_LRI_ (root primordia/100 cortical cells) in transgenic plants indicates increased root branching (Figure 2F). These findings demonstrate that SDG8i activity promotes both primary root growth and lateral root initiation.

### Overexpression of SDG8i affects auxin homeostasis

 The spatial distribution of auxin controls many aspects of plant growth and development [26]. The synthetic auxin response reporter construct *DR5-GUS*, has been used in many studies to examine the patterns of auxin distribution [27]. To examine the effect of SDG8i activity on auxin levels and distribution, the SDG8i transgenics were crossed with plants containing the *DR5-GUS* reporter construct [28] and the progeny analyzed for histochemical GUS activity [22]. While GUS activity appeared slightly elevated during early growth stages in SDG8i transgenics when compared with controls, the difference in staining was quite pronounced at the four leaf stage with transgenics showing much more GUS activity in the root tips and the vascular tissue of shoots and high level GUS activity at the leaf margins (Fig. S5). These results suggest that endogenous auxin levels are elevated in SDG8i plants. 

### SDG8i plants exhibit salt and freezing tolerance

**Figure 3:**
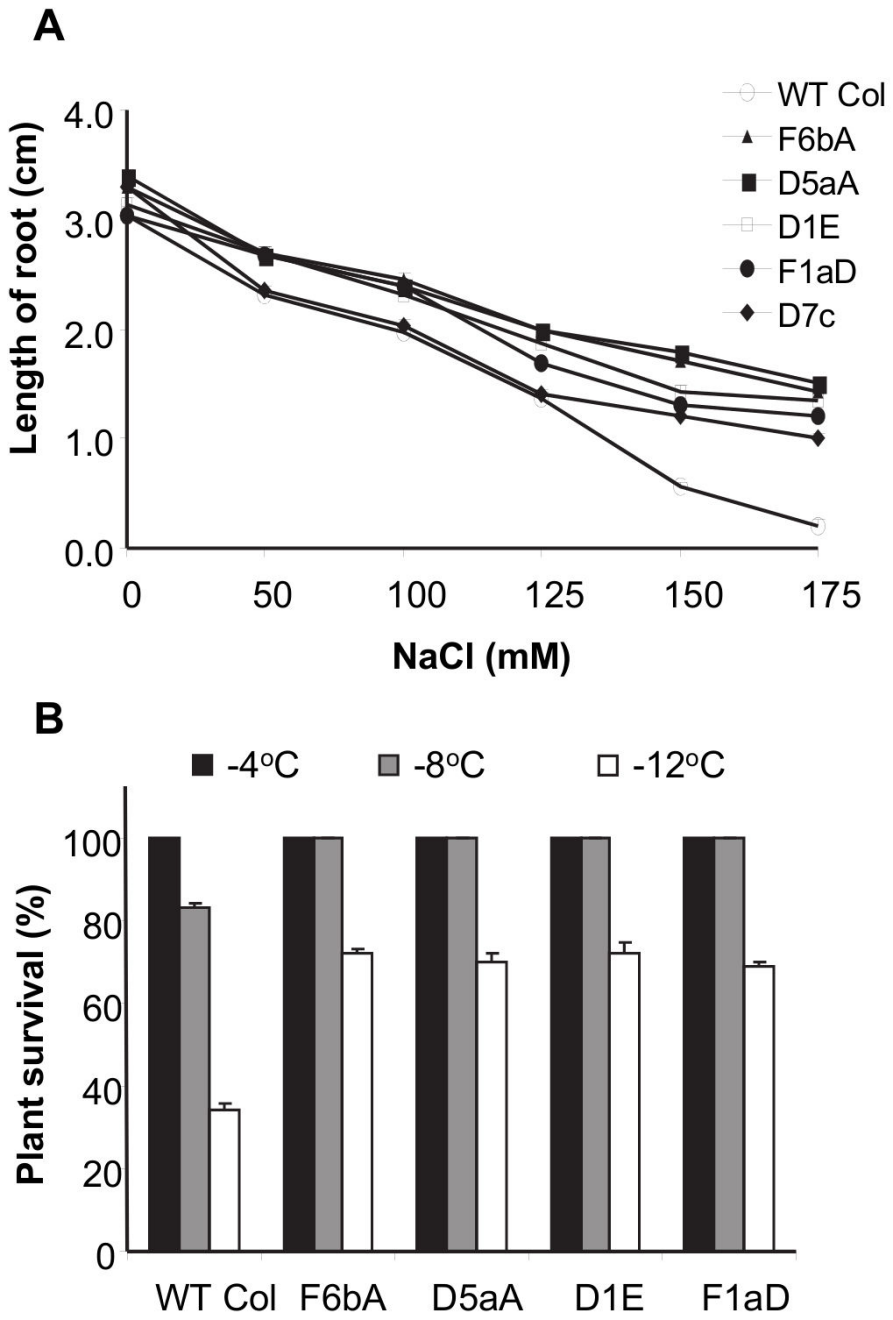
Salt and freezing stress tolerance of SDG8i lines. (A) Effect of salt on the root growth of SDG8i lines and wild-type Col-0. Root growth was measured after 7 days following transfer to NaCl. Values are the means ± SE of 10 replicates. (B) Freezing tolerance of SDG8i lines and wild-type Col-0. Plants were exposed to freezing at -12°C after cold-acclimation at -1°C for 16 hours. Survival was scored 7 days after the plants were returned to normal growth conditions at 22°C. Values are the means ± SE of 3 replicates.

 A comparison of the response of SDG8i transgenics and wild-type plants to growth on high salt media (Figure 3A) showed no substantial difference at salt concentrations below 100 mM after 7 days growth. However, at 150 mM NaCl all transgenic lines showed 2-3 times more primary root growth than that of wild-type plants (*p*<0.001). At 175 mM NaCl wild-type plants showed severe inhibition of root growth (Figure 3A and Fig. S6A) whereas the root growth of transgenic lines was 5-7 times that of wild-type plants (*p*<0.001). 

 A freezing tolerance test was also performed on the transgenic and wild-type control plants (Figure 3B and Fig. S6B). All transgenic and control wild-type Col-0 plants exposed to -4°C freezing stress survived. At -8°C, all SDG8i transgenic lines survived, whereas the survival rate of wild-type Col-0 plants was 83% (*p*<0.01). At -12°C, control wild-type Col-0 plants were affected greatly with a survival rate of only 34%, whereas the survival rate of the transgenic plants ranged from 69-72% (*p*<0.01). 

### SDG8i transgenics are drought tolerant

**Figure 4:**
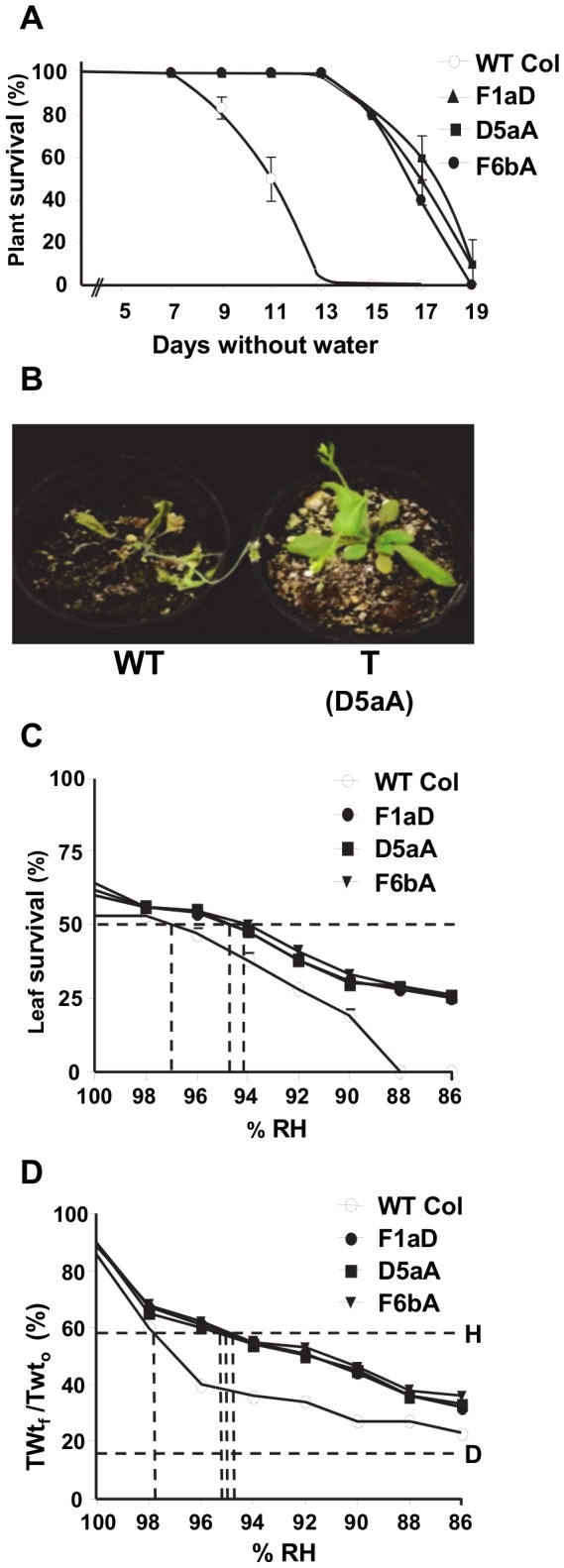
Drought tolerance of SDG8i plants. (A) Plants were deprived of water, re-watered at intervals and monitored for recovery as described in the text. Values are the means ± SE of 2 replicates. (B) Wild-type Col-0 (WT) and a transgenic plant (T) after 13 days without water. (C) Leaf survival and (D) PDT survival curve [% of final saturation weight to the initial turgid weight ratio] showing the effect of decreasing relative humidity on cell survival of SDG8i and WT shoots. PDT value is the water potential (expressed as the corresponding equilibrium relative humidity at 20°C) at which 50% of cells are dead after 3 days vapor equilibration over CaCl_2_ osmotica. Line D represents the lowest value of TWt_f_/TWt_0_ reached by comparable shoots killed by chloroform vapor. Line H is the 50% survival point halfway between line D and 100% TWt_f_ /TWt_0_.

 The response of SDG8i transgenics to water-deficit was compared to that of wild-type plants. Plants were kept fully watered by sub-irrigation until the 6-7 leaf pre-flowering stage. Water was then withheld over a period of 3 weeks, then the plants were re-watered and recovery monitored after 24 hours. In the wild-type Col-0 plants, wilting was visible after 7 days and after 13 days of drought all control plants were severely dehydrated and showed signs of senescence, whereas all SDG8i transgenic plants were still green and healthy (Figure 4A, B). The survival rate of wild-type plants was 50% after 11 days (*p*<0.001) and by the thirteenth day had dropped to zero. On the other hand, the survival rate of transgenic plants was 100% after 13 days and then reduced to 50% after 17 days. All the transgenic plants were severely dehydrated on day 18 and they did not recover after one further day of drought treatment. 

 To quantify the increase in drought tolerance conferred by SDG8i expression, a measure of protoplasmic drought tolerance (PDT; determined as the percent lowest relative humidity at which 50% of leaf cells survive) was obtained. The PDT of wild-type plants, as determined by both the subjective method (Figure 4C) and the objective method (Figure 4D) was ~97-98% RH (~-2.8 to -4.2MPa). This indicates that *Arabidopsis* is very sensitive to water deficit, which is not surprising in the thin tender leaves on well-watered greenhouse plants of an ephemeral species. The three transgenic lines gave closely similar data to each other in both methods. Their PDT value of ~94-95% RH (~-7.0 to -8.4MPa) is considerably better than the value obtained for wild-type plants, indicating a substantial improvement of PDT (*p*<0.05). 

### SDG8i activity delays dark-induced senescence and acts downstream of ABA

 To test the effect of SDG8i activity on senescence, detached leaves from wild-type and transgenic plants were floated on ddH_2_O in the dark and chlorophyll degradation monitored over several days.

**Figure 5:**
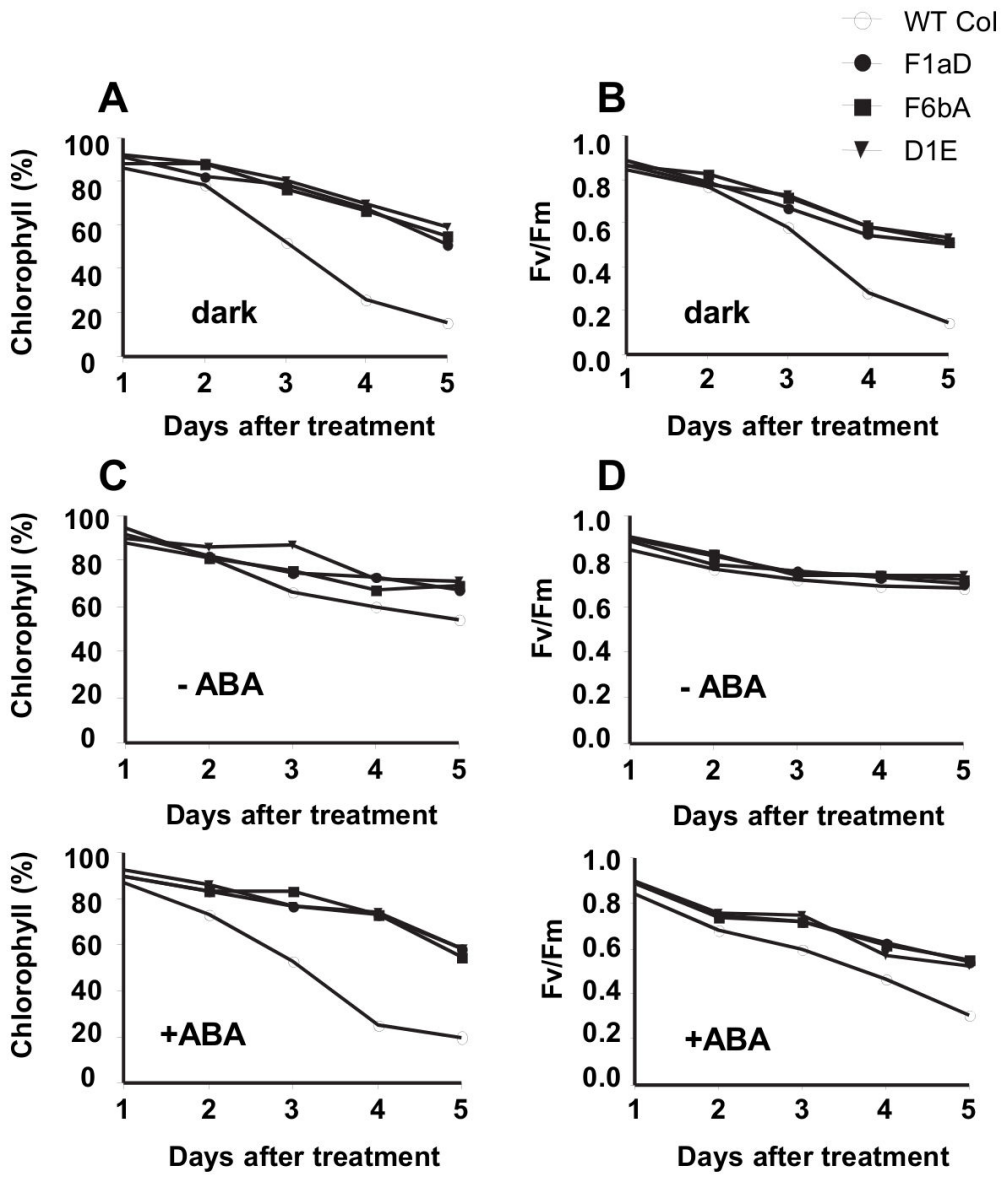
Dark- and ABA-induced senescence in wild-type and SDG8i leaves. (A) Chlorophyll content and (B) photochemical efficiency in dark-treated detached leaves of WT and SDG8i plants. (C) Chlorophyll content and (D) photochemical efficiency in detached leaves of WT and SDG8i plants under continuous light in the absence or presence of 50 µM ABA.

 After five days in darkness the chlorophyll content and the photochemical efficiency (F_v_/F_m_) values of wild-type plants decreased to around 20% (*p*<0.001) (Figure 5A, B) and the leaves turned completely yellow. In the SDG8i transgenic lines the leaves remained green after five days, retaining about 55-60% of their chlorophyll, with F_v_/F_m_ values of about 52-53% of the original reading (*p*<0.05) (Figure 5A, B). These results indicate that SDG8i transgenic plants exhibit reduced dark-induced senescence.

 To test the relationship between SDG8i activity and the senescence-promoting phytohormone ABA, we repeated the assay under continuous light with, and without, the exogenous hormone (Figure 5C, D). Over the five days incubation in continuous light without ABA, the chlorophyll content of control wild-type leaves reduced to 54%, whereas SDG8i transgenic leaves retained around 71% of their chlorophyll content. The F_v_/F_m_ values for both transgenic and control plants were not significantly different under these conditions. Upon treatment with ABA, the chlorophyll content of wild-type leaves decreased rapidly from day two down to 20% by day five (*p*<0.001) (Figure 5C). On the other hand, the SDG8i transgenic leaves retained about 75% of their chlorophyll up to day four and then reduced to around 58% by day five (*p*<0.05) (Figure 5C). The F_v_/F_m_ value of SDG8i transgenics was also substantially higher than control plants on day five (*p*<0.005) (Figure 5D). These results show that SDG8i expression reduces ABA-induced senescence and suggests that it functions downstream of ABA. 

### *SDG8i* encodes a UGT that glycosylates GR24 *in vitro*

**Figure 6:**
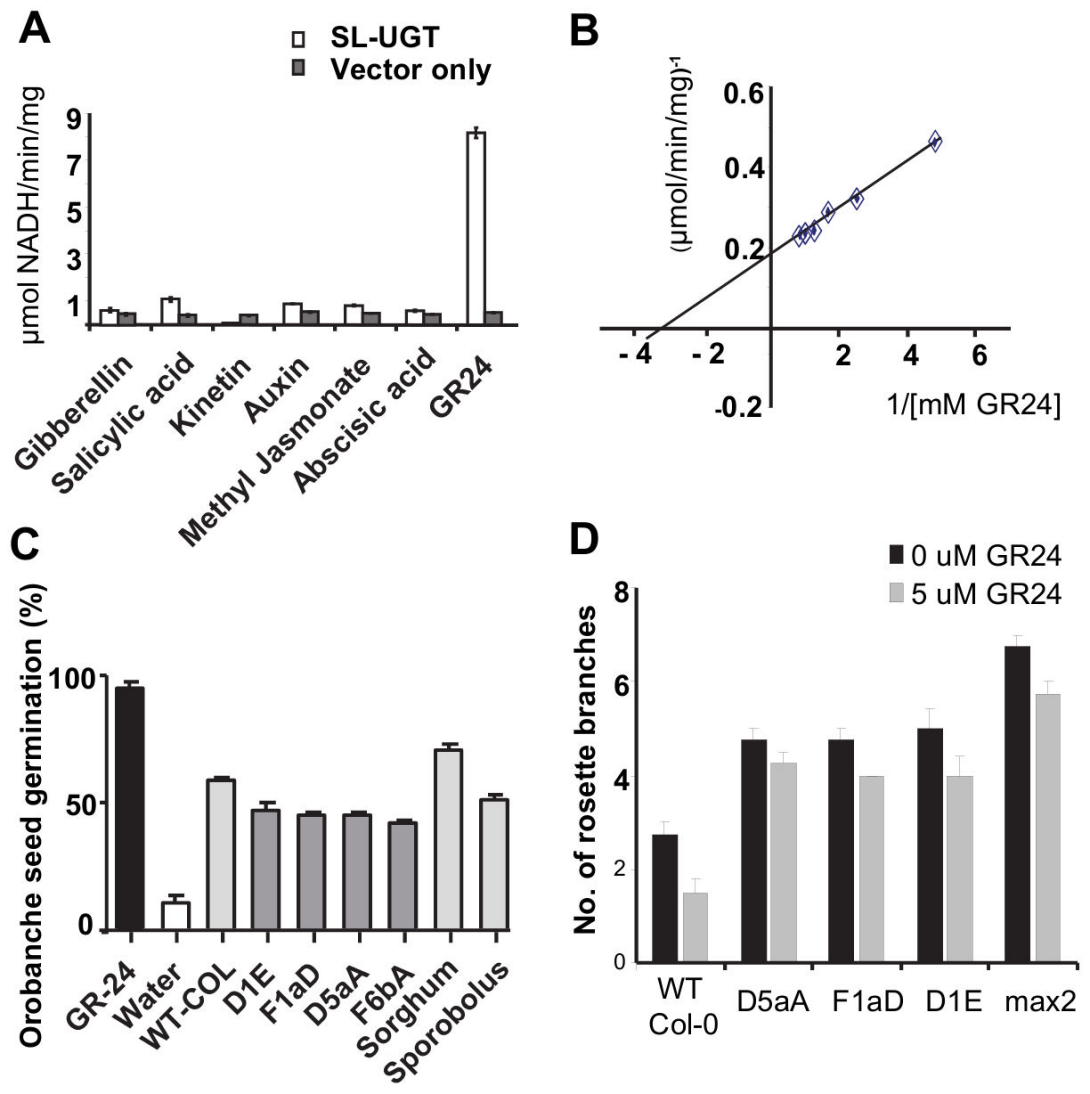
Analysis of the enzyme activity of *SDG8i in vitro*. (A) The glycosyltransferase activity of SDG8i recombinant protein extract using the plant hormones gibberillin (GA3), salicylic acid, kinetin, auxin (IAA), methyl jasmonate, ABA([±]-*cis*, trans-abscisic acid and the synthetic strigolactone analogue GR24 as substrate (p < 0.05). The coupled enzyme assay [9] was conducted at pH 7.4 using 25μg of protein extract with a final substrate concentration of 1mM. Rates were calculated per mg total protein extract. GR24 was obtained from Chiralix B.V. Nijmegen,The Netherlands. All other hormones were obtained from Sigma St. Louis, MO. (B) A Lineweaver-Burke plot of SDG8i activity with varying concentrations of GR24 indicating a Km of 0.349mM and a Vmax of 5.67 μmole/min/mg. Rates were calculated per mg total protein extract. (C) Level of stimulation of germination of *Orobanche* seeds in response to root induction by wildtype Col-O and SDG8i transgenic *Arabidopsis*, sorghum and *S. stapfianus* seedlings in vitro. The percent germination was calculated by counting the number of seeds having an emerged radicle. Values are the means ± SE of 5 replicates. (D) Effect of GR24 (0 or 5 µM) on bud outgrowth of wild-type Col-0, SDG8i transgenic and max2 *Arabidopsis* plants. Plants were treated with GR24 on the rosette shoot meristem, axillary buds and leaf axils every third day for 20 days and the number of branches was counted after 43 days. Data are means ± 4 replicates.

 The phenotype of the transgenic plants suggests that SDG8i UGT activity is influencing hormone homeostasis. To investigate the catalytic function of SDG8i, the UGT activity of *Nicotiana benthamiana* leaf extracts infiltrated with an actin (*AtACT2*)-promoter driven *SDG8i* construct, using a viral-based system [19], was tested against a number of plant hormones as substrates and compared with UGT activity in extracts infiltrated with a vector-only control. The substrates used were chosen for their known ability to affect plant growth and stress responses. The SDG8i extract showed substantial glycosylation activity of the strigolactone analogue GR24 (Figure 6A) with a K_m_ of 0.349 mM and a V_max_ of 5.67 μmole/min/mg (Figure 6B). The activity observed with the other substrates showed very little increase over background endogenous NADH oxidase activity. Similarly, the vector-only control extract showed no substantial activity above background with GR24 or with any of the other substrates. The results indicate that *SDG8i* encodes a glucosyltransferase with *in vitro* activity against a strigolactone-like compound*.*

### The stimulation of *Orobanche* germination is reduced in *Arabidopsis* expressing SDG8i

 Root exudates from *Arabidopsis* can stimulate the germination of *Phelipanche ramosa* (*Orobanchaceae*) [29]. The *Orobanchaceae* germination bioassay has been used extensively to determine the level of strigolactone secreted from host plant roots [30]. To determine if SDG8i activity has an effect on the root secretion of these stimulus signals that are required by parasitic plants to germinate, four SDG8i transgenic *Arabidopsis* lines were compared with wild-type Col-0 *Arabidopsis* plants for the ability to stimulate *Orobanche* germination in axenic culture (Figure 6C and Fig. S7). GR24, *Sorghum bicolor* and *S. stapfianus* were included as controls. Germination of 95% was achieved by treatment with GR24, while 11% germination was observed in the water control. The levels of *Orobanche* seed germination stimulated by sorghum and *S. stapfianus* was 71% and 52% respectively. The 60% germination of *Orobanche* seeds by wild-type Col-0 plants was significantly higher than that of the all the transgenic lines tested (*p*<0.001) with SDG8i transgenic lines stimulating 42-47% germination. The transgenic line, F6bA, showed the highest reduction in *Orobanche* germination (30%) compared with the wild-type Col-0 *Arabidopsis* control. These results suggest that SDG8i activity may be reducing the level of strigolactone germination stimulants secreted from the roots of *Arabidopsis.*

### GR24 inhibition of shoot branching is reduced in SDG8i transgenic plants

The branching response of wild-type Col-0, SDG8i transgenic and the branching mutant *max2 Arabidopsis* plants were tested by the application of GR24 to leaf axils and axillary buds before and during bolting as described by Gomez-Roldan et al. [31] in order to see whether they are able to respond to SL-mediated shoot branching inhibition (Figure 6D). In wild-type Col-0 plants, shoot branching decreased by 55% in response to GR24 compared to control plants (p<0.001). However, in all SDG8i transgenic plants and *max2* mutants, application of GR24 decreased shoot branching by only 10-15% compared to untreated plants (p<0.001) with branching remaining elevated over treated or untreated wild-type Col-0 plants. 

## Discussion

### The phenotypic changes mediated by overexpression of *SDG8i* suggests the enzyme may affect strigolactone-related processes

 Several UGTs, capable of influencing the biological activity of plant hormones via glycosylation, have been isolated [9-15]. The ability of SDG8i recombinant protein to glycosylate the synthetic strigolactone analogue GR24 *in vitro*, combined with the phenotypic changes observed in SDG8i overexpressing lines, suggests that SDG8i glucosyltransferase activity may be effecting the strigolactone signalling pathway in the transgenic plants. As well as mediating the interactions of host plants with symbiotic fungi or parasitic plants, the well-conserved strigolactone hormone signaling system contributes to environmental regulation of plant growth. Strigolactones have been shown to respond to environmental cues, such as low nutrient conditions (Pi, N), to interact with abscisic acid (ABA), and to be involved in growth-related auxin and ethylene activity [32-35]. Strigolactones are carotenoid-derived terpenoid lactones that are synthesized mainly in the roots and can be transported in the xylem sap to the shoots [29]. In some plant species, mutations in strigolactone production or perception genes have been shown to delay flowering time, reduce senescence and decrease root mass [36,37]. Root exudates containing strigolactones can stimulate parasitic *Striga* and *Orobanche* plant germination and hyphal branching of symbiotic arbuscular mycorrhizal fungi [38]. Strigolactones have been shown to regulate root growth in response to phosphate and/or carbohydrate availability [39,40]. In the shoots, increased strigolactone levels inhibit tiller formation or lateral bud outgrowth [29,41] and mutations in the strigolactone biosynthesis and signaling pathway are often associated with a hyperbranched, dwarfed phenotype [32,33]. 

 Several characteristics of the SDG8i ectopic expression lines mimic those of the strigolactone MAX mutants, including hyperbranching, reduced senescence and interactions with auxin and ABA [33,34,37]. However, the reduced stimulation by SDG8i plants of *Orobanche* germination, indicative of diminished strigolactone levels [30], is modest and the SDG8i enhanced shoot and root growth phenotypes are opposed to those of SL-deficient mutants which are usually dwarfed. While the moderate affinity of SDG8i for GR24 could be expected given the artificial nature of the substrate and assay conditions, it is clear that the SDG8i enzyme in *Arabidopsis* is affecting the bioactivity of an endogenous growth-related compound/s. MAX2/ORE9, the F-box leucine-rich repeat signaling protein is expressed in the vasculature and plays multiple roles in different parts of the plant, not all of which are dependent on the activity of the SL branching signal generated by MAX3 and MAX4 [42]. Several of the processes associated with MAX2 activity appear to be negatively affected in SDG8i plants. In addition to auxin interactions and suppression of branching, MAX2 acts to promote leaf senescence [37] and repress hypocotyl elongation in the light [43] and is also involved in oxidative stress and drought responses [44,45]. Hence the phenotype of SDG8i transgenic plants (which includes elongated hypocotyls) appears to be consistent with a reduction in the activity of MAX2. The reduced branching inhibition in SDG8i plants by GR24 application also supports this contention. This would suggest that crossing SDG8i transgenics with MAX3 or MAX4 mutants would be unlikely to lead to suppression of the mutant phenotypes, however crossing SDG8i transgenics with plants overexpressing MAX2 may be informative in elucidating the mode of action of SDG8i. The genetic analyses with strigolactone mutants and an examination of expression levels of MAX pathway genes are underway, however, full comprehension of the mode of SDG8i action requires identification and structural analysis of the endogenous target metabolite/s. Identification of the metabolite/s could substantially enhance our knowledge of how environmental influences regulate plant growth. Since glycosylation is widely accepted as having a role in bioactivity, transport and stability of many growth regulators [7], an effort to identify an endogenous enzyme with similar SDG8i activity in *Arabidopsis* and other plants may be worthwhile, although substrate specificity of UGTs is not necessarily reflected in gene phylogeny and *in vitro* substrate screening of recombinant UGTs is usually required to identify functionally similar UGTs between species [8]. 

### SDG8i activity may impede the stress-related growth retardation response

 Plant productivity, even when plants are growing under near-ideal conditions, can be limited by the innate response of plants to seasonal influences or short-term stress events. These responses often involve reduced growth and/or reallocation of resources into less desirable growth patterns. The imposition of stress below toxic levels can elicit apoptosis or growth redistribution within minutes to diminish stress exposure [46]. The pathways integrating environmental stressors with inhibition of internal growth-related processes in plants is not well understood [47] and the introduction of stress-related genes to increase constitutive stress tolerance often correlates with diminished growth. In contrast, SDG8i activity promotes cell expansion and division in shoots and roots under both stress and non-stress conditions. In the resurrection grass *S. stapfianus*, SDG8i gene activity, following transcript accumulation in desiccated tissue, may be associated with leaf regeneration, which is more than twice as rapid following a dehydration/rehydration cycle as in a well-watered plant [4]. The enhanced growth exhibited by SDG8i transgenic *Arabidopsis* plants under stress, suggests that SDG8i activity may impede the growth retardation response which would normally occur under these conditions. A reduction in the ability of a plant to initiate morphogenic changes in response to environmental influences could be expected to compromise the survival of wild species over long-term stress events. However, substantial growth advantages may be conferred in the context of crop cultivation with ample mineral nutrition and shorter-term stress events. The phenotype of the SDG8i transgenic plants provide an important example of the potential of bioengineering to enhance shoot-root biomass and seed yield in plants, whilst simultaneously conferring substantial improvements in salt, cold and drought resistance. The ability to utilize SDG8i activity to control stress-related and seasonal morphogenic responses in crops could represent a substantial advance in domestication of food plants.

### The *SDG8i*-mediated drought-tolerance improvement may be associated with inhibition of the drought-related senescence program

 Considerable research effort has been put into increasing crop production utilizing the isopentenyltransferase (*IPT*) gene to increase cytokinin biosynthesis. Increased cytokinin levels can delay drought-induced senescence and allow retention of higher chlorophyll levels under water-deficit [48-50]. The problems associated with lower yields due to altered source sink distribution appears to have been overcome by utilizing a senescence associated receptor kinase (SARK) promoter to drive the *IPT* gene [51-53]. Use of the *SDG8i* gene presents an alternative approach to reducing drought-induced senescence and potentially has the additional benefit of increasing yield under non-stress conditions. The two-fold improvement in the water potential survived by the SDG8i transgenic lines, compared with the untransformed wild-type *Arabidopsis* plants, is considerable and is similar to the maximum ‘drought hardening’ reported for a crop plant droughted for 3 weeks [54]. The reduced senescence displayed by transgenic SDG8i plants suggests that SDG8i activity may also be associated with inhibition of dehydration-induced senescence programs, a phenomenon that occurs in the younger leaves of the desiccation-tolerant *S. stapfianus* plant. In non-resurrection plants, leaf senescence is thought to be an efficient strategy for surviving water-deficit by reducing canopy size and transpiration [55]. Interestingly, SDG8i transgenic plants showed no sign of senescence after 13 days without watering yet survived much better than wild-type plants. The droughted wild-type plants ceased vegetative growth, the leaves senesced rapidly, and the plants produced an inflorescence of 2-3 cm before dying. The transgenics on the other hand remained green and healthy. Some transgenics formed one or more extra rosette leaves and a reproductive meristem during the drying stage, but did not appear to accelerate reproductive development. The reduced senescence, in combination with the larger leaves, may be related to the higher seed yield of the SDG8i transgenic plants. 

 The use of SDG8i to generate a more robust productive plant with enhanced growth and stress resistance, combined with the benefit of reduced stimulation of parasitic seed germination, could herald a novel approach for increasing food production in agriculturally important crop plants.

## Supporting Information

Figure S1 **RNA gel-blot analysis showing the presence of *SDG8i* transcripts in SDG8i transgenic lines.**
(TIFF)

Figure S2 **Flower morphology, seed development and growth characteristics, of wild-type Col-0 plants (WT) and SDG8i plants (T) growing at 21°C in LD conditions.**
(TIFF)

Figure S3 **Growth characteristics of SDG8i plants growing in short days.**
(TIFF)

Figure S4 **Palisade cells in the second fully expanded rosette leaves of wild-type Col-0 and SDG8i transgenic lines.**
(TIFF)

Figure S5 **Histochemical staining showing differential GUS activity at various stages of development typical of wild-type Col-0 plants and SDG8i transgenic (D5aA) plants crossed with *DR5::GUS Arabidopsis* seedlings.**
(TIFF)

Figure S6 **Salt and freezing stress tests of wild-type Col-0 (WT) and SDG8i transgenic (T) *Arabidopsis* seedlings in vitro.**
(TIFF)

Figure S7 **Stimulation of germination of *Orobanche* seeds.**
(TIFF)
