# Supplementary figures and images for: Increased Biomass, Seed Yield and Stress Tolerance Is Conferred in Arabidopsis by a Novel Enzyme from the Resurrection Grass Sporobolus stapfianus That Glycosylates the Strigolactone Analogue GR24
Source: PLoS One. 2013 Nov 5;8(11):e80035. doi: 10.1371/journal.pone.0080035 (PMC3818285; doi:10.1371/journal.pone.0080035)

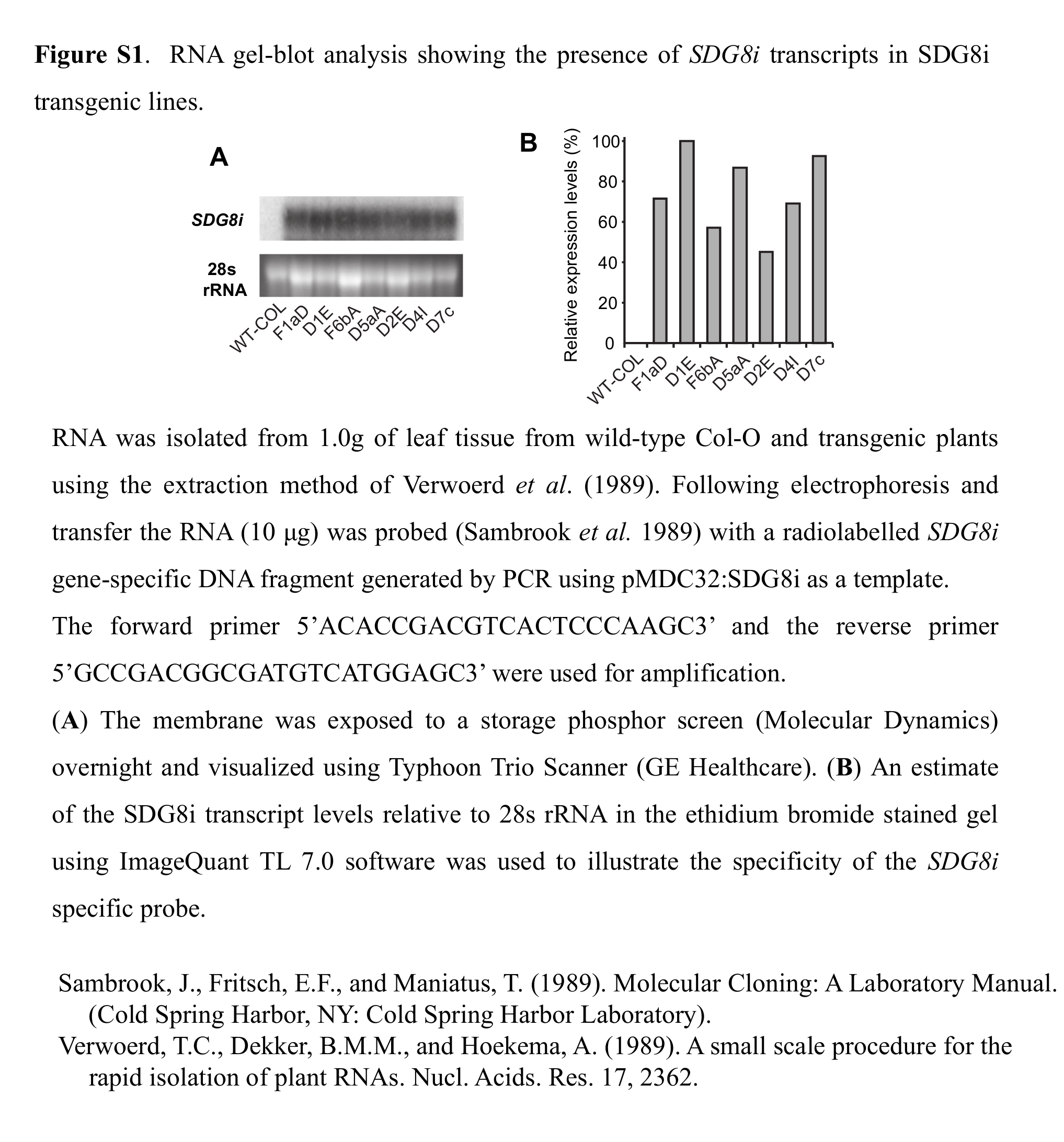

Supplement: Figure S1 — RNA gel-blot analysis showing the presence of SDG8i transcripts in SDG8i transgenic lines. (TIFF) [file pone.0080035.s001.tiff]

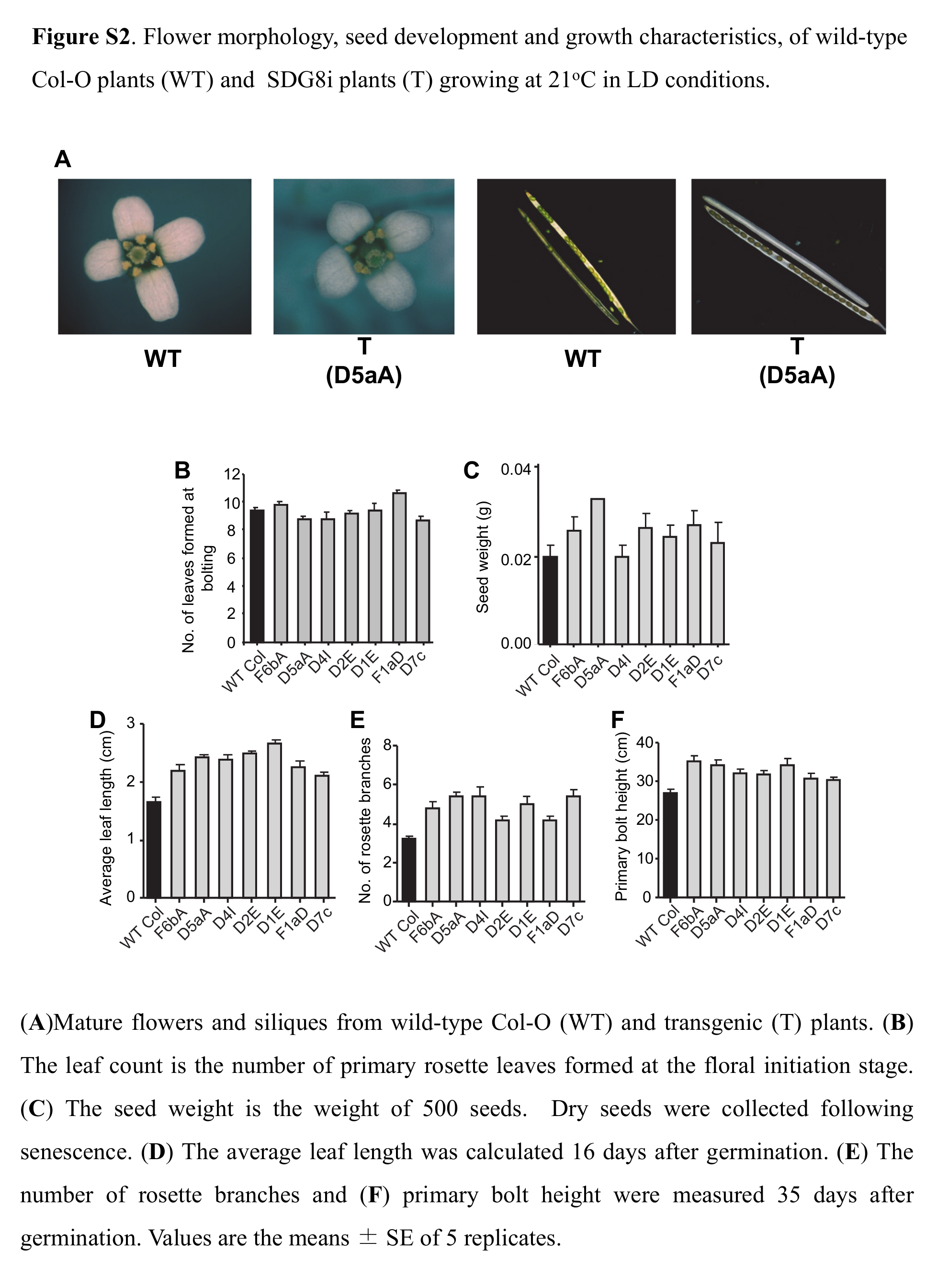

Supplement: Figure S2 — Flower morphology, seed development and growth characteristics, of wild-type Col-0 plants (WT) and SDG8i plants (T) growing at 21°C in LD conditions. (TIFF) [file pone.0080035.s002.tiff]

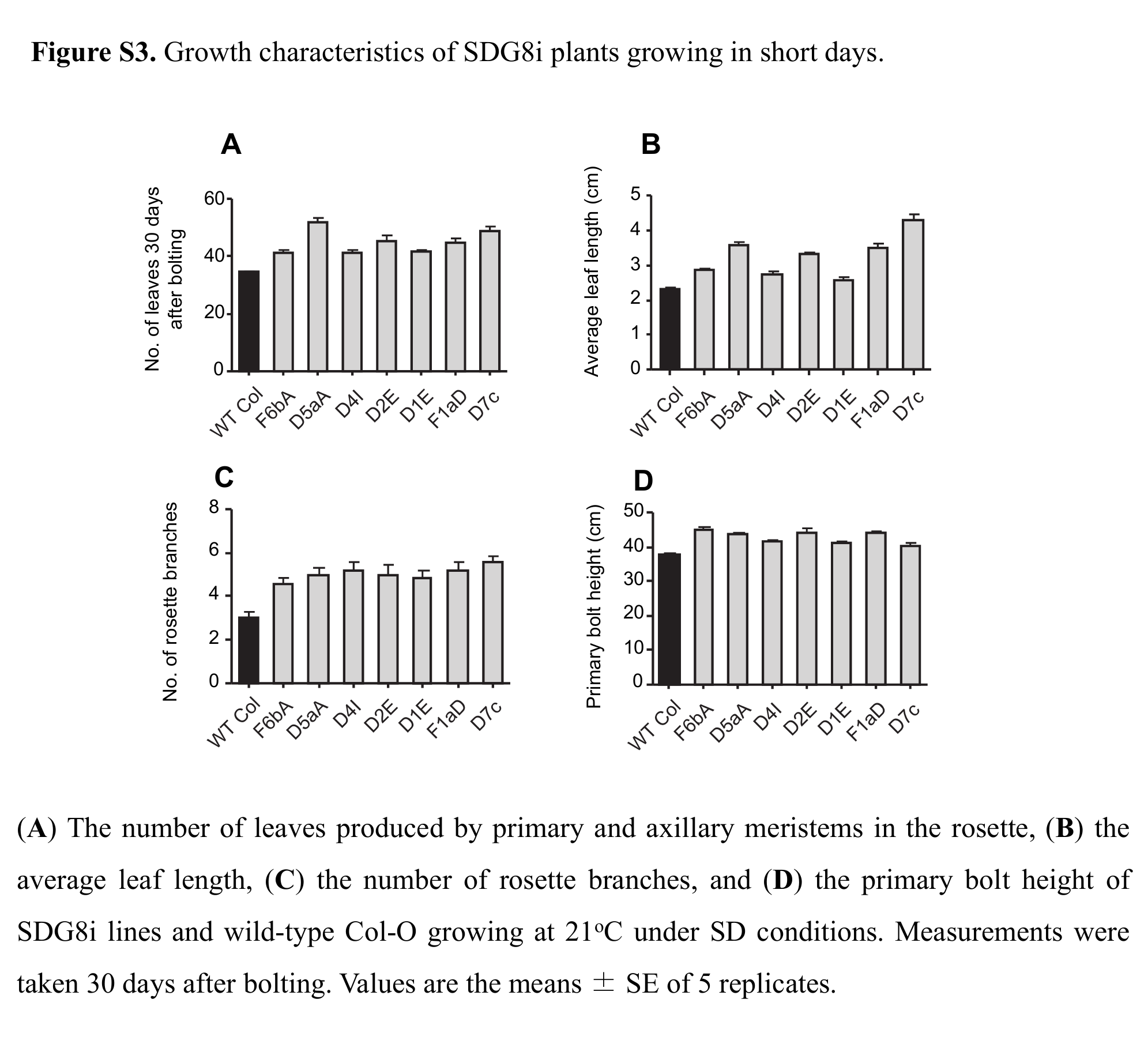

Supplement: Figure S3 — Growth characteristics of SDG8i plants growing in short days. (TIFF) [file pone.0080035.s003.tiff]

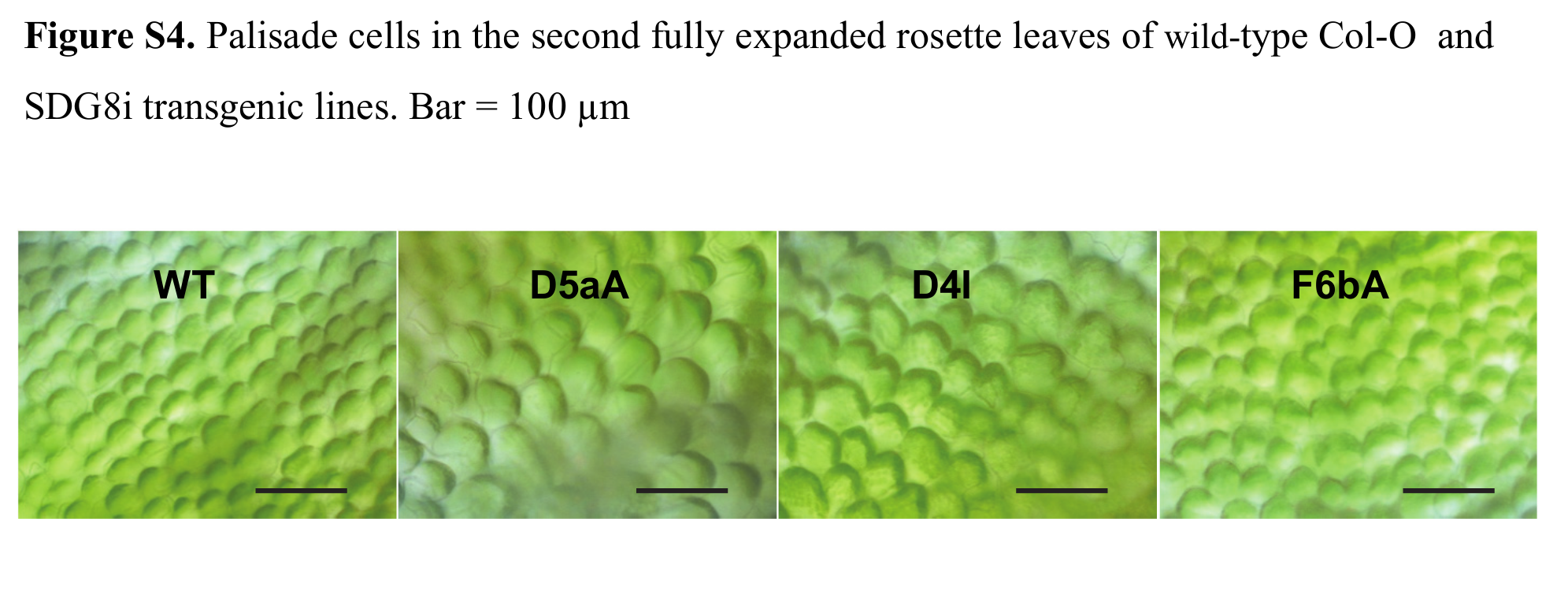

Supplement: Figure S4 — Palisade cells in the second fully expanded rosette leaves of wild-type Col-0 and SDG8i transgenic lines. (TIFF) [file pone.0080035.s004.tiff]

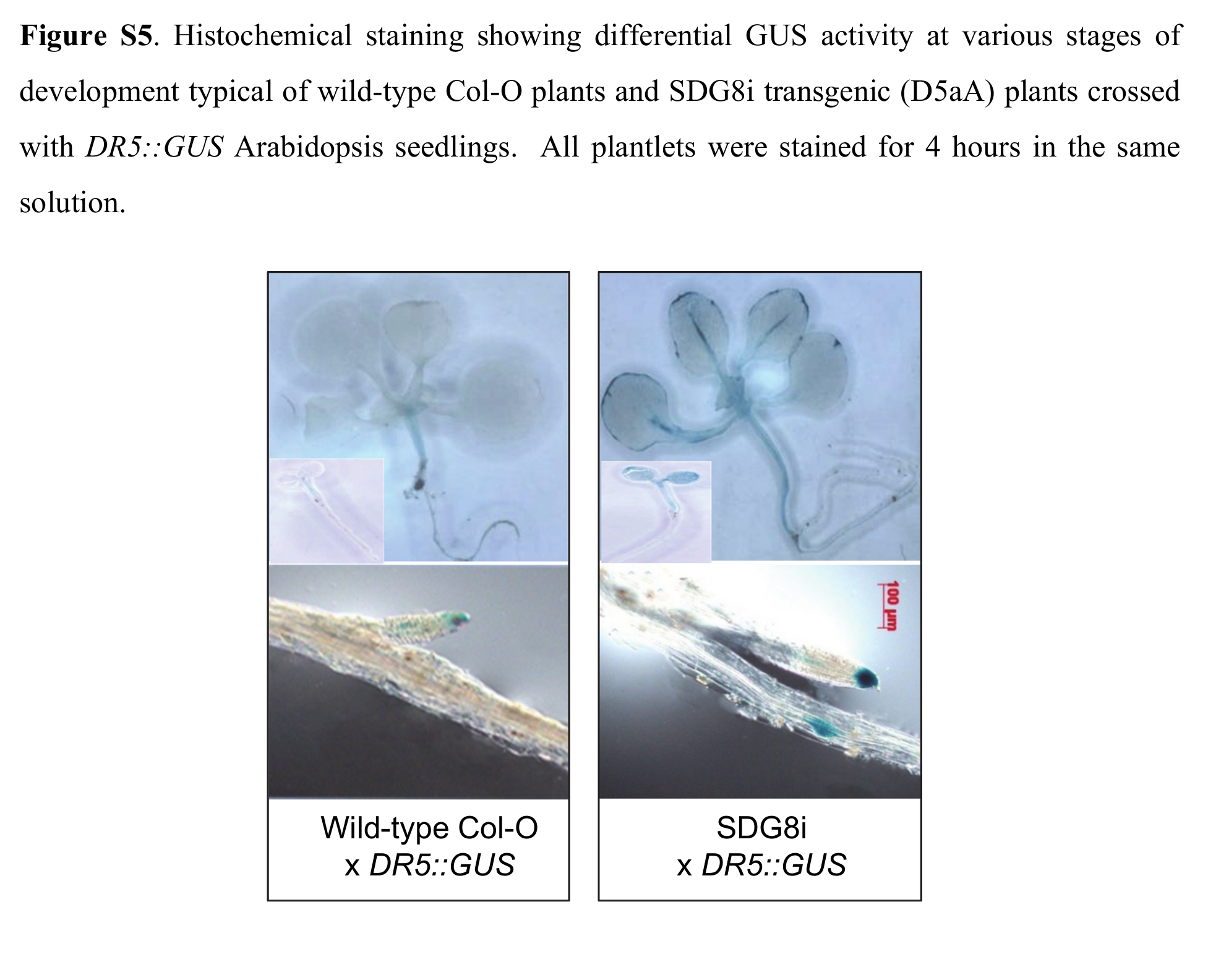

Supplement: Figure S5 — Histochemical staining showing differential GUS activity at various stages of development typical of wild-type Col-0 plants and SDG8i transgenic (D5aA) plants crossed with DR5::GUS Arabidopsis seedlings. (TIFF) [file pone.0080035.s005.tiff]

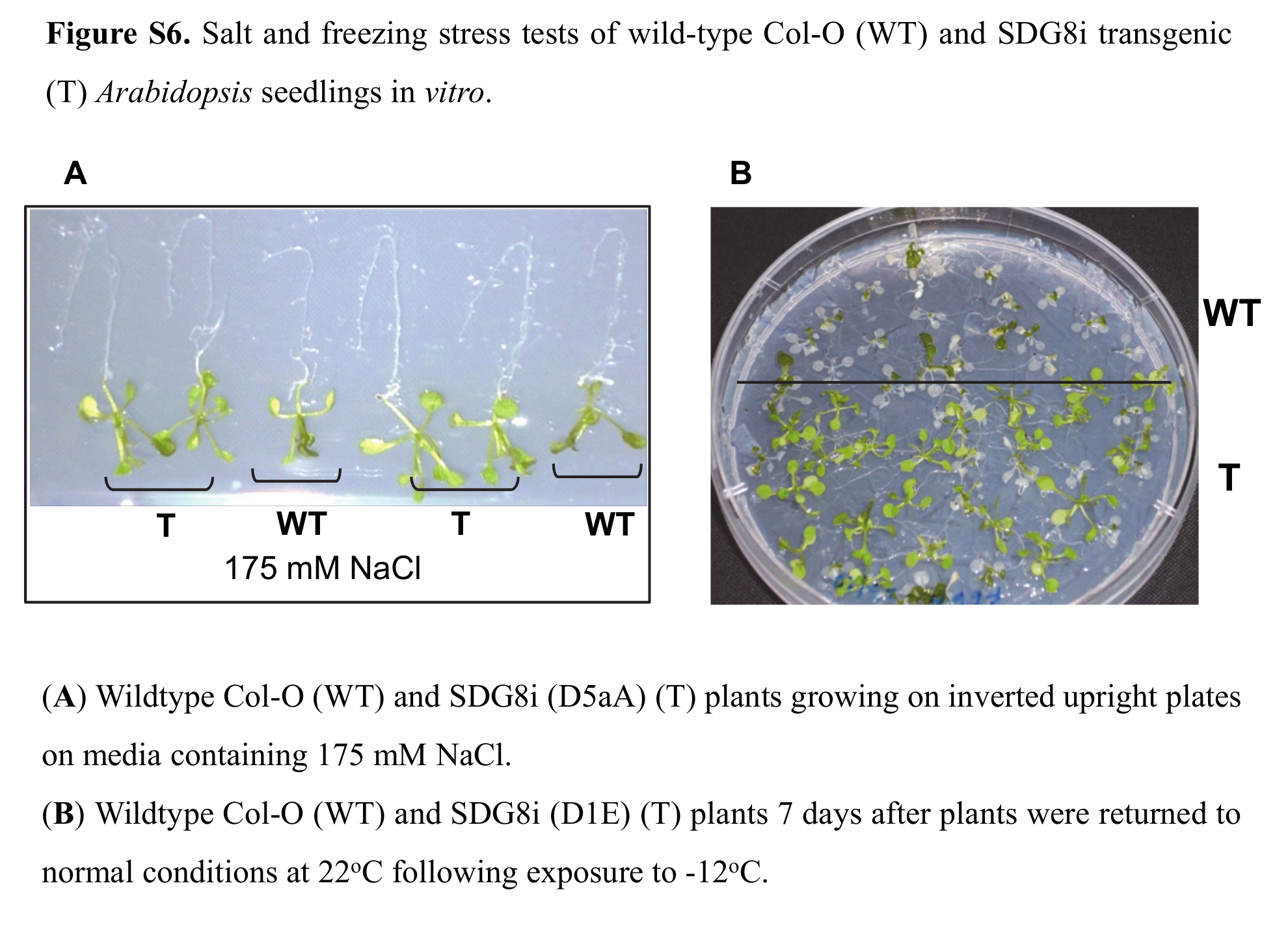

Supplement: Figure S6 — Salt and freezing stress tests of wild-type Col-0 (WT) and SDG8i transgenic (T) Arabidopsis seedlings in vitro. (TIFF) [file pone.0080035.s006.tiff]

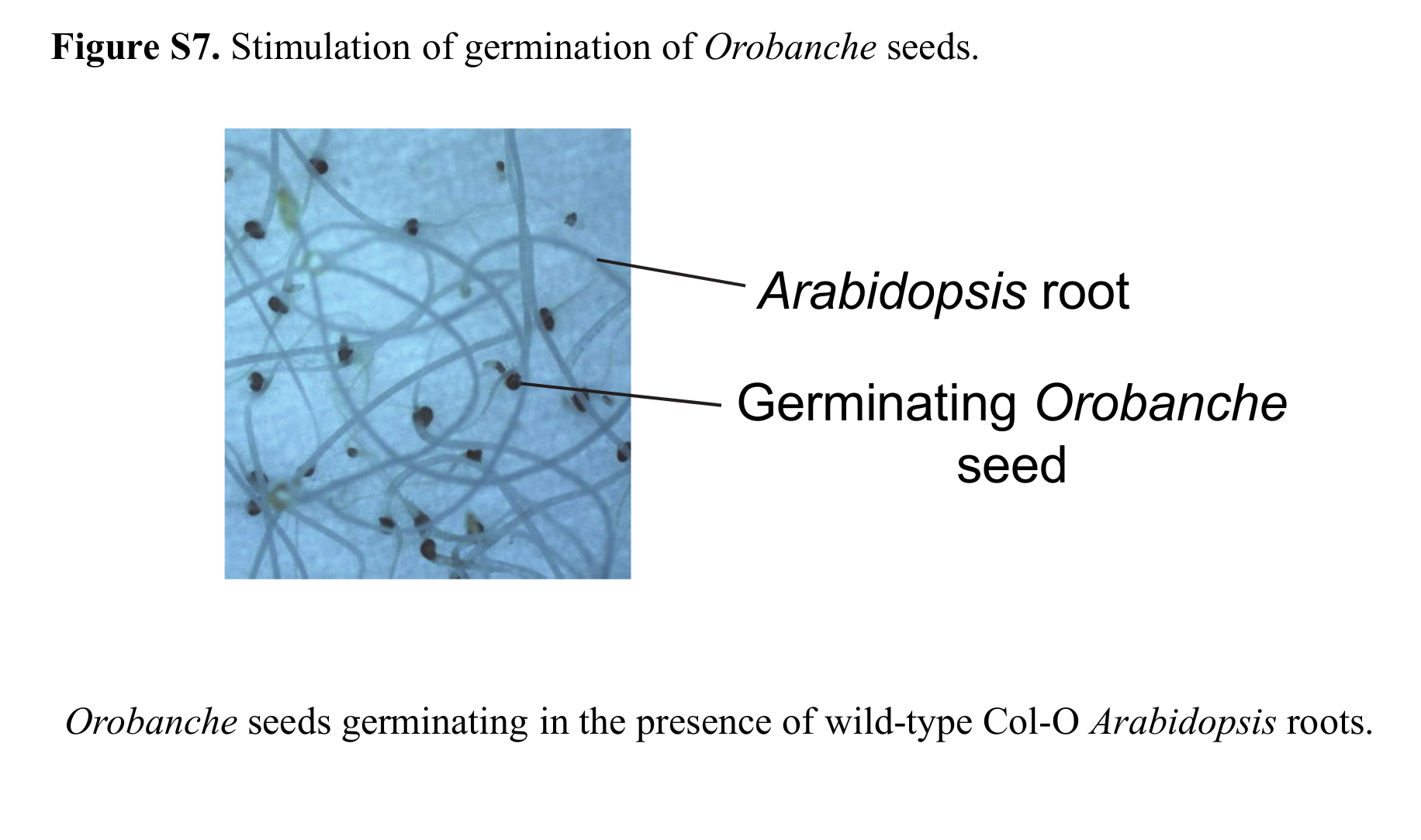

Supplement: Figure S7 — Stimulation of germination of Orobanche seeds. (TIFF) [file pone.0080035.s007.tiff]
